# International Analysis of the Nutritional Content and a Review of Health Benefits of Non-Dairy Plant-Based Beverages

**DOI:** 10.3390/nu13030842

**Published:** 2021-03-04

**Authors:** Winston J. Craig, Ujué Fresán

**Affiliations:** 1 Center for Nutrition, Healthy Lifestyles, and Disease Prevention, School of Public Health, Loma Linda University, Loma Linda, CA 92354, USA; 2 eHealth Group, Instituto de Salud Global Barcelona (ISGlobal), 08036 Barcelona, Spain; ujuefresan@gmail.com

**Keywords:** plant-based non-dairy beverages, plant-based milks, nutrient composition, fortification, calcium, vitamin D, vitamin B12, protein, sugar

## Abstract

Concerns about environmental impact and sustainability, animal welfare, and personal health issues have fueled consumer demand for dairy alternatives. The aim of this study was to conduct a cross-sectional survey of plant-based non-dairy beverages from three different continents (USA, Australia, and Western Europe) to assess their nutritional content and health profile. A total of 148 non-dairy beverages were analyzed from the nutrition label and ingredients listed on the commercial package or from the information located on the website of the manufacturer or retailer. The different types of beverages were extracts of nuts or seeds (*n* = 49), grains (*n* = 38), legumes (*n* = 36), coconut (*n* = 10), and mixed blends (*n* = 15). On average, the plant-based beverages generally scored well in terms of not containing high levels of sodium, saturated fat, or calories. Over half of the beverages were fortified with calcium to levels equal to or greater than that of dairy milk. The protein content varied from 0 to 10 g/serving. Levels of vitamin D and B12 fortification were quite low. Consumers should be informed of the nutritional profile and potential health benefits of plant-based dairy alternatives as the nutritional content can vary greatly between the different types of beverages.

## 1. Introduction

A visit to the dairy section of any major supermarket in the West will reveal the vast array of non-dairy plant-based beverages based upon a wide variety of plant products. The popularity of these products has dramatically increased over the past decade. A significant number of people living in Western countries limit or avoid dairy milk altogether for many reasons including milk protein allergies, lactose intolerance, personal and environmental health concerns, or from a desire to support their vegan lifestyle [1]. Globally the dairy alternatives market was reported to be worth $US12.1 billion in 2018 and is expected to reach $US25.1 billion by the end of 2026 [1]. The non-dairy milk industry in the US grew 61% from 2012 to 2018 [2]. Millennials, who make up nearly one quarter of the US population, are reported to be the largest group to consume non-dairy milk with 77% of them buying the beverages regularly [3].

Soy was the first dairy alternative beverage on the market and enjoyed substantial popularity. However, consumer confidence in soy fell significantly when unfavorable internet stories surfaced about the high isoflavone content of soy and its possible association with cancer. However, the use of soy has not been documented to be associated with an increased risk of breast cancer [4]. Almond milk then grew in popularity before articles on the web touted the so-called health benefits of coconut and coconut beverage [5]. With the steady popularity of almond beverage, additional beverages made from other nuts soon appeared on the market. Today, we have more than 20 non-dairy plant-based beverages from which to choose. These plant-based beverages can be made from nuts (e.g., almonds, cashews, hazelnuts), coconuts, grains (rice, oats), legumes (soy, peas), seeds (flax, hemp, sesame, chia), and fruits (banana). Almond, soy and coconut non-dairy beverages have been the recent market leaders, with the sales of oat-based beverages now experiencing a surge [2]. Preferences for the beverages vary depending upon a number of factors such as the flavor, price, packaging, taste, nutritional profile, and whether the product is organic, and free of genetically modified (GM) ingredients [6,7].

Previous investigators have analyzed non-dairy beverages against dairy milk as a nutritional standard, with a special focus mostly on women and children. They conclude that the non-dairy beverages should not be considered nutritionally equivalent to dairy milk [8,9,10,11,12]. In contrast to this, 69% of consumers believe that non-dairy plant-based beverages are healthy for their kids [13].

Typically, a serving of plant-based dairy alternative beverage supplies less than 140–150 calories. This represents only 7–7.5% of a 2000 calorie intake for a day. A serving of 2% (reduced fat) milk would provide 124 calories. A non-dairy beverage is not usually considered a major source of calories. However, there are certain nutrients that are normally provided from the use of milk. It is imperative that a plant-based dairy alternative be adequately fortified with these essential nutrients, namely calcium, vitamins D and B12. For a vegetarian, these three nutrients may not be easily obtained in their diet in sufficient amounts without the presence of dairy milk. For the vegans, it is especially important since their diet is typically marginal in these three nutrients [14].

Consumers, for health reasons, are often concerned about the level of sodium, saturated fat and sugars in the plant-based dairy alternative beverages. These nutrients can have an adverse effect upon one’s cardiovascular [15,16,17] and metabolic health [18], and body weight [19]. Since the plant-based beverages are often used in place of dairy milk, some consumers desire the protein level to approximate the level in dairy milk, that is 8 g protein/serving. The presence of dietary fiber in a processed food is considered a positive thing. Its presence promotes gastrointestinal and cardiovascular health, and improves glycemia and insulin sensitivity in both diabetic and non-diabetic individuals [20]. The addition of prebiotic fibers, such as chicory root extract, to some beverages is considered a healthy addition since they may also enhance immune function [20].

The purpose of the present study was to conduct a cross-sectional survey of plant-based non-dairy beverages to assess the nutritional content and health profile of the plant-based beverages. The fortification level of calcium, vitamins D and B12 for each beverage was determined. In addition, the chemical form of calcium fortification was also documented since various calcium salts are known to have different bioavailability. Both calcium carbonate and tricalcium phosphate are commonly used, yet they have been shown to have different levels of absorption [21]. The levels of protein, sodium, saturated fat, sugar, and dietary fiber in the plant-based beverages were also examined in an effort to measure the nutritional quality and health profile of each plant-based beverage.

In this study we were not only interested in the important nutritional and health pros and cons for using the different beverages but also to discover how the different types of beverages (grain, nut, seed, coconut and legume based beverages) varied in composition. Are the products typically fortified in a consistent pattern? Are there differences between the various products available from different parts of the Western world? Our results should help consumers make better informed choices.

## 2. Materials and Methods

The nutritional contents of 148 plant-based dairy milk alternatives were analyzed. This included 60 beverages (22 brands) from the Western USA, 48 beverages (11 brands) from Australia, and 40 beverages (16 brands) from Western Europe (in particular UK, France, and Spain). The beverages were selected, from October to December 2020, from those commonly available in selected supermarkets on the 3 continents, including Safeways (USA), Woolworths (Australia), Waitrose (UK) and Carrefour (Western Europe). Store beverages were photographed and additional beverages were added from the website of the retailer. All plant-based beverages observed in the stores and shown in the appropriate websites were considered except those beverages that had incomplete information on the nutrition label.

The nutritional content and ingredients were recorded from the nutrition label on the commercial package or from the information located on the website of the manufacturer or retailer. The nutrients per serving size which were available on all packages included calories, fat, saturated fat, sodium, dietary fiber, total sugars, protein, and the important micronutrients calcium, vitamin D, and vitamin B12. The calcium salt used in fortification and the form of gum (dietary fiber) used were also noted. The median values of the nutrients were calculated for each type of beverage and for the beverages grouped from each of the three continents. The median levels of fortification were calculated (for calcium, vitamin D, and vitamin B12 separately) for all beverages as well as separately for those that are actually fortified.

The nutritional value of each beverage was rated according to the following criterion: calcium, vitamin D and vitamin B12 content of at least 20% of Daily Value (DV)/serving; and at least 5 g of protein/serving. The health qualities portrayed by the ingredients were determined by the following criteria: not more than 5 g of total sugars/serving; not more than 1 g of saturated fat/serving; not more than 120 calories/serving; not more than 115 mg sodium/serving; and at least 1.5 g of dietary fiber/serving.

The US Dietary Guidelines specify, as a general guide, that 5% DV or less of a nutrient/serving is considered low, while 20% DV or more of a nutrient/serving is considered high [22,23]. In the USA the DV for calcium is 1300 mg, vitamin D is 20 mcg, vitamin B12 is 2.4 mcg, sodium is 2300 mg, protein is 50 g, added sugars is 50 g, saturated fat is 20 g, and dietary fiber is 28 g [23]. For our analyses we considered a 20% DV (high level) as an adequate fortification for calcium, vitamin D and vitamin B12. For sodium and saturated fat we accepted that beverages should not exceed 5% of their DV (a designated low level), i.e., 115 mg for sodium (5% of 2300 mg) and 1 g of saturated fat (5% of 20 g). For European beverages, the sodium content was calculated from the given salt value using the formula that 2.5 g salt equals 1000 mg sodium. We suggest that beverages have at least 5% of DV for dietary fiber (approx. 1.5 g), at least 10% of the DV/serving for protein, and no more than 10% DV/serving for sugars. Ten percent was chosen as a mid-stream number between the 5% DV (low value) and the 20% DV (high value). This gave us a minimally acceptable level of 5 g protein/serving, and a level of 5 g sugars/serving to allow for some sweetening but not an excessive amount.

### Statistical Analysis

The percentage of non-dairy plant-based beverages having been fortified with specific micronutrients (i.e., calcium, vitamin D and vitamin B12) and with specific nutritional requirements per serving (i.e., at least 5 g protein, no more than 1 g saturated fat, no more than 5 g total sugars, no more than 120 calories, no more than 115 mg sodium and at least 1.5 g dietary fiber) were detailed. Descriptive statistics (median and interquartile range) was stated for products by region and by type of beverage, as the normality of data distribution was firstly verified through the Kolmogorov-Smirnov test and rejected. The Kruskal–Wallis test for independent samples was used to evaluate variability in calories and nutrients of interest per serving among groups, followed by Bonferroni adjustment for multiple comparisons. The statistical analysis was performed through the IBM SPSS Statistics 27.0.1.0 (Armonk, NY, USA: IBM Corp.) with the significant level set at *p* < 0.05.

## 3. Results

The plant-based beverages analyzed were based upon almonds (*n* = 33), soy (*n* = 29), oats (*n* = 23), rice (*n* = 13), coconut (*n* = 10), cashews (*n* = 7), pea protein (*n* = 7), hazelnuts (*n* = 3), macadamia (*n* = 3), flax (*n* = 2), quinoa (*n* = 2), hemp (*n* = 1), and the following mixtures: almond and coconut (*n* = 6), pea and almond (*n* = 2), oats and pea (*n* = 1), pea, almond and cashew (*n* = 1), rice and hazelnut (*n* = 1), rice and coconut (*n* = 1), oats and walnut (*n* = 1), hazelnuts, walnut and almond mix (*n* = 1), and soy and rice (*n* = 1).

The analyses of the nutritional composition and health profile of the 148 plant-based non-dairy beverages are summarized in Table 1, Table 2, Table 3, Table 4, Table 5, Table 6 and Table 7. From the data shown in Table 1 we see that the beverages are more commonly fortified with calcium (78%) than with vitamins D (53%) or B12 (41%). Fortification generally occurs more frequently for products sold in the USA than in Australia and Europe. This is most marked for vitamin D. Frequency of vitamin D fortification is especially poor in Australia (21%) compared with the USA (82%). In addition, vitamin B12 fortification is below 50% in all 3 regions.

A level of 20% DV or more is considered high for a nutrient by the US Dietary Guideline standards [23]. When all the beverages were examined against this standard we found that most of the products that were fortified reached that 20% DV standard for calcium (70%), while B12 fortification achieving the 20% DV level were only 35–40% of the beverages, and vitamin D fortification to the 20% DV standard were remarkably low (24%) (Table 1). In fact, less than 10% of the beverages sold in Europe and Australia contained vitamin D at the 20% DV level. Even after eliminating the non-fortified beverages from the statistical calculations, the median levels of vitamin D in all 3 regions were low (9–22% DV) (Table 2).

Mean values of calories and four selected nutrients (saturated fat, protein, sugars, and sodium) in the beverages are tabulated in Table 3. The beverages as a whole appeared modest in calories (median: 93 kcal/serving), sodium (median: 100 mg/serving), and sugar content (median: 4.6 g/serving), and low in saturated fat (median: 0.5 g/serving) and protein (median: 1.8 g/serving). Twenty-four (16%) of the beverages were labeled as unsweetened. No differences in the protein content or sugar level was detected among countries (*p* = 0.055 and *p* = 0.056, respectively). The energy content of Australian beverages appeared higher than the American ones, and their sodium content higher than that of the European beverages. Table 4 and Table 5 reveal the number of beverages with a healthy profile, as defined by their saturated fat, sugar, sodium, calorie, protein, and dietary fiber levels/serving. The data is broken down by region (Table 4), and by type of beverage (Table 5). While fewer than 30% of the beverages overall contained significant levels of dietary fiber (at least 1.5 g per serving), 55% of the beverages contained no more than 5 g sugars/serving, 64% contained no more than 115 mg sodium/serving, 74% contained no more than 120 calories/serving, and 87% contained no more than 1 g saturated fat/serving. By contrast, only 26% of the beverages contained at least 5 g protein/serving. Only the beverages based on soy or pea protein had levels of protein in excess of 5 g/serving. In addition, the beverages based on soy, oats, and macadamia were likely to have higher levels of dietary fiber. Only beverages based on pea protein had more than half their products with sodium levels over 115 mg/serving. Most of the beverage types had more than half of their products with 5 g or less of sugars/serving. Beverages based upon rice, hazelnuts, and oats were the exceptions.

Protein levels varied considerably among the various types of beverages (Table 6). While 1 in 4 beverages contained at least 5 g protein/serving (Table 4), the protein levels varied from 0.1 g/serving for coconut to 8–9 g/serving beverages having either soy or pea protein as their base (Table 6). The grain-based beverages ranged from 0.8 g protein/serving for rice to 2.0 g/serving for oats. Nut-based beverages contained protein levels of 1.0–1.3 g/serving (Table 6).

The analyses described below are concerned with issues of fortification. Supplemental Tables S1 and S2 show selected nutrient values for all of the beverages, both fortified and non-fortified products, by world region (Table S1) and by beverage type (Table S2). Calcium fortification varied among the beverages 2-fold (Table 7). Beverages based upon coconut and cashews had a median value of 16% DV; oats and rice beverages 25% DV; soy and pea-based beverages 30% DV; and almond beverages were 35% DV of calcium/serving. While 60% of beverages overall were not fortified with vitamin B12, the lack of B12 was especially noticeable in almond-based beverages (7/33 fortified), cashew-based (3/7 fortified), oats-based (10/23 fortified), rice-based (6/13 fortified), and beverages from mixed sources (2/14 fortified). Of the fortified beverages, soy, almond and cashew beverages had a median value of 50% DV of vitamin B12/serving while pea, coconut and oat-based beverages had a median value of 35–39% DV, and rice beverages 25% DV for vitamin B12 content/serving (Table 7). The lack of vitamin D fortification by beverage type was very similar to that seen for vitamin B12. Almond, almond-coconut blend, cashew and rice beverages had less than 50% of their products fortified with vitamin D. Among the fortified beverages, the lowest vitamin D levels were seen in hazelnut, coconut and almond beverages (9–12% DV/serving), and the oats, rice and soy beverages (15% DV/serving) (Table 7).

We noticed that there was a fortification pattern observed among the manufacturers. Some brands appear fortified with calcium and the two vitamins (D and B12) while other brands consistently lack 2 or all 3 nutrients.

Only 115 of the 148 products were fortified with calcium, and of those reporting the calcium salt used for fortification, tricalcium phosphate was the most commonly used (44%), followed by calcium carbonate (38%). Sixteen percent of the products were fortified with both tricalcium phosphate and calcium carbonate. Calcium chloride and calcium hydroxide were each used once in a beverage. Many products contained no added fiber and those that had added fiber components often contained 2 to 3 different sources. Most of the dietary fibers added to beverages provide functional properties (as a stabilizer/thickener) rather than for any stated nutritional value. An exception to this was any beverage labeled as prebiotic. The website of the food company claimed these products improved gut health. Of the 82 products reporting the addition of fiber, gellan gum (83%) was the most popular addition, followed by locust bean gum (26%), xanthan gum (20%), and guar gum (13%). Seven reported carrageenan, five reported chicory root extract (those labeled as “prebiotic”), four reported gum arabic (acacia gum). Others reported the addition of citrus fiber, sodium alginate, marine algae, or cellulose.

In addition to the nutrients listed above, a few of the beverages showed the presence of other nutrients: riboflavin (*n* = 22), vitamin E (*n* = 8), thiamine (*n* = 7), vitamin A (*n* = 4), zinc (*n* = 3), and selenium, magnesium, copper, pyridoxine, folic acid (1 product each).

## 4. Discussion

### 4.1. Nutrients of Importance

Along with the growing surge of interest in non-dairy plant-based beverages, there has appeared a few regional studies on these plant-based beverages from different parts of the world. As new products continually enter the market place a comprehensive survey of these products is timely. In our analysis across 3 continents, we place our results in juxtaposition with the other studies done in the past 5 years that analyzed at least 25 products grouped in at least 5 types of beverages [8,11,12]. Those 3 papers all were focused on how the nutritional profile of the beverages compared with dairy milk. We focused on calories and some 3 critical nutrients (calcium, vitamin D and B12) as well as 5 others that reflected more on the health quality of the beverage (such as sodium, sugar, and saturated fat). A major emphasis in our study focused on the level of fortification as it related to the 3 critical nutrients. We also wanted to review some other issues that are often overshadowed in the discussions that non-dairy beverages do not compare favorably with the nutritional profile of dairy milk, and hence it is ill advisable to give these non-dairy beverages to toddlers and children as a replacement for dairy milk [8,9,10,11]. A major reason for this is the observation that very few non-dairy plant-based beverages are fortified with the necessary vitamins and calcium [11,12]. These reports have highlighted the reduced level of protein, calcium and certain micronutrients in the alternative beverages. Typically, Western diets rely upon milk to contribute substantial levels of three nutrients, vitamin D, calcium, and vitamin B12. Non-dairy plant-based beverages should be fortified with these nutrients to prevent potential deficiencies.

The low incidence of vitamin D and B12 fortification (53% and 41%, respectively) of the plant-based beverages (Table 1) is unfortunate since these beverages can provide a significant amount of these vitamins for vegetarians. Even among those beverages that were fortified, levels of fortification were inadequate in more than 60% of cases for vitamin B12 and three-quarters of the cases for vitamin D (see Table 1). The low level of vitamin D fortification in Australian products (21%) is noteworthy (see Table 1), given the reported high prevalence of vitamin D deficiency in Australia [24]. While an Australian research group did not report vitamin D levels in their 115 beverages, they do report that beverages contained little or no vitamin B12 content [8]. From the ingredient lists provided by a Canadian research group it appears that 8/17 (47%) of the beverages were fortified with vitamin D and 8/17 were fortified with vitamin B12 [10]. However, in both cases no mention is made of the degree of fortification. In the analysis of 45 Swiss plant-based beverages, the authors reported only 14% were fortified with one or more of vitamins D, B2, or B12 [11]. However, when the beverages were fortified the amounts were equivalent to that in dairy milk [11]. In the large Italian study the authors focused on macronutrients rather than on vitamin and calcium fortification. In addition, they were interested in how the nutritional content of the beverages was impacted by organic certification, nutritional and health label claims [12].

In our findings for the beverages that were vitamin B12 fortified, rice beverages had the lowest content of B12, while soy, pea, cashew and almond beverages had the highest content of vitamin B12 (Table 7). For the beverages that were vitamin D fortified, almond and coconut beverages had the lowest content of vitamin D, while pea protein had the highest.

Calcium fortification of a non-dairy beverage can typically range from 100–450 mg/serving compared to 300 mg/serving for dairy milk. We found calcium fortification to be much better (almost 80% of the non-dairy beverages) compared to vitamins D and B12 fortification. In addition, about 70% of those fortified with calcium achieved the level of 20% DV/serving (designated as a high nutrient content) (Table 1). In fact, 82 (55%) of the beverages were fortified to levels equal to or greater than the calcium level of dairy milk. This compares favorably with the Canadian group [10] which reported 11/17 or 64% of the non-dairy beverages with levels of calcium similar to dairy milk, while the Australian group reported only one-third of the non-dairy beverages with similar levels of calcium as dairy [8]. Sousa et al. reported that almond, soy, and quinoa showed the highest level of calcium among the beverages they tested [11]. This was similar to our finding that almond, soy and pea-based beverages (they did not test pea-based beverages) had the highest levels of calcium (Table 7). Lowest levels of calcium occurred with the coconut and cashew beverages.

Different calcium salts can be used for fortification of a beverage. Calcium carbonate and tricalcium phosphate were the most commonly used calcium salts we observed in the fortification of dairy milk alternatives. The two salts were used almost equally (40–45% of the time) while 1 in 6 beverages used both salts. The absorption of calcium from calcium carbonate is reported to be equivalent to that from dairy milk, while tricalcium phosphate is significantly less than that of dairy milk [25].

Many of the plant-based beverages are considered poor sources of protein. The median value for protein in our study (1.8 g/serving) compared favorably with that of the Italian study (0.7 g/100 mL or 1.75 g/serving) [12]. Only those non-dairy plant beverages made from soy or pea protein had a protein level (approx. 8–9 g/serving on average) (Table 6) comparable with that of dairy milk (8 g/serving). A total of 21 beverages (14%) had a protein level exceeding that of dairy milk. All of these contained either soy or pea protein. Rice and coconut based beverages have negligible amounts of protein (0–1 g/serving), while almond and other tree nuts have 1–1.5 g/serving. Some of the newer blended varieties add pea protein to boost the protein level. Those beverages with a protein content below 5 g protein/serving would not be considered ideal for growing children. These concerns have been expressed elsewhere [9,11]. A survey found that a significant number of Americans would be encouraged to drink or drink more non-dairy beverages if they had more protein [13].

In addition, it is important that consumers regularly read the product label, since formulations change with time and levels of fortification were observed to vary significantly between brands and even within different types of the same brand (for example, sweetened, unsweetened, vanilla, original, classic, etc.). A number of large supermarket chains have store brands of non-dairy plant-based beverages that typically sell for less than national name brand products. Unfortunately, many of these store brand products have little or no fortification.

### 4.2. Healthy Profile

While many of the beverages (over 70%) had low protein levels (less than 5 g/serving) and the level of vitamins D and B12 fortification could be improved, there were some redeeming factors. All of the beverages, except the coconut-based beverages, had very low saturated fat levels (no more than 1 g/serving). In addition, over 60% of the beverages had low levels of sodium (no more than 5% DV or 115 mg/serving), and no beverage exceeded 230 mg of sodium/serving (10% DV). We found that pea-based beverages had the most sodium added, while coconut, hazelnut, and soy averaged the lowest levels of sodium. The Swiss group and Australian groups found coconut to have the lowest sodium levels [8,11]. The way that other research teams grouped the beverages together did not permit us to make any further comparisons.

More than half of the beverages (55%) contain low to modest levels of sugars (less than 5 g/serving), while almost 3 in 4 beverages had a modest level of calories (no more than 120 calories/serving). The amount of added sugars (usually cane sugar) is one of the important issues considered when making a choice for a dairy alternative [26]. While some people may claim that these plant-based beverages have high levels of salt and sugar, this is not really the situation, in most cases. While 101 beverages (68%) examined in this study were labeled as sweetened, it is interesting to note that only 25 (17%) of the beverages had high level of sugars (at least 10 g/serving or 20% DV/serving). While some of the beverages could benefit from an improvement in their nutritional content, there are choices available to the consumer (who reads labels carefully) to make healthy choices. One should carefully read the label to learn how many grams of sugar occur in a serving. The beverages flavored with vanilla or chocolate tended to be amongst the sweetest varieties. Our data also agreed with the Swiss, Italian and Australian research groups [8,11,12] that found the grain beverages (such as rice and oats) had more sugar than other beverages types. Coconut, cashew, pea, and almond based beverages had the lowest sugar levels, a finding not inconsistent with another report [8].

Most plant-based milks contain about 2.5 to 4.5 g fat/serving. The level of fat in dairy milk varies depending upon the type of milk, ranging from 0.2 g/serving in non-fat milk to 8 g fat in whole milk. The level of fat in plant-based non-dairy milks is comparable to that of 1% and 2% (reduced fat) dairy milk (2.4–4.9 g fat/serving). However, the fat in dairy milk is predominantly saturated fat while in plant-based milks the fat is largely heart-healthy unsaturated fat. For soy, flax and hemp seed-based milks the fat is predominantly polyunsaturated fat while for rice milk and the nut milks (almond, cashew, hazelnut and macadamia) it is predominantly monounsaturated fat. Oats has an equal mix of polyunsaturated and monounsaturated fat as well as a healthy level of soluble fiber. In contrast, the fat in coconut milk (4–4.5 g/serving) is at least 90% saturated fat [27]. The saturated fat significantly raises LDL and total cholesterol levels in clinical trials [28]. Even though coconut fat has a good content of short and medium chain fatty acids, coconut is not as healthy as other vegetable oils [28]. The ability of coconut oil to raise one’s HDL cholesterol level may somewhat offset the atherogenic effect of its very high saturated fat content [29]. The presence of phenolic antioxidants in virgin coconut oil has also been suggested as a factor that may partially ameliorate the negative effect of the high saturated fat content of coconut oil [30]. However, we are unaware to date that any food company uses virgin coconut oil to make their coconut milk. We note that plant-based non-dairy beverages contain no cholesterol, while dairy milk contains 5–34 mg/serving depending upon the level of fat in the dairy milk.

While most of the plant-based milks have 1–2 g fiber/serving, some (such as coconut and cashew) have negligible amounts, while dairy milk has none. In earlier times, plant milks used carrageenan, derived from a red seaweed. It is perfectly safe and has been approved for use as a thickening and gelling agent in food [31]. A chemically altered form of carrageenan was reported to be associated with a low risk of cancer [32]. Due to this concern, companies have used alternate thickening agents. Nevertheless, seven beverages we examined used carrageenan. Gellan gum, a water-soluble fiber, is now the most commonly used gum throughout the world as a thickening agent, emulsifier and stabilizer. Four out of 5 beverages we surveyed contained gellan gum. In plant based beverages it helps to keep plant protein suspended in the milk. Other gums that were commonly used as thickening agents for plant-based milks we surveyed included locust bean (or carob) gum (26%), xanthan gum (20%) and guar gum (13%). All of these water-soluble gums are useful for managing glycemia and hypercholesterolemia [20,33,34].

Another factor to consider which has health implications would be the glycemic index (GI) of the plant-based beverages. A diet comprised of lower GI foods is associated with a lower risk of obesity, diabetes, and cardiovascular disease [35]. A low GI (<55) and medium GI (56 to 69) food is recommended, especially for those who wish to better regulate their blood glucose levels [35]. Compared to dairy milk with a GI of 47, most of the non-dairy alternatives had a GI ranging from 50 to 60 (macadamia 50, cashew 53, soy 53, quinoa 53, hazelnut 56, almond 57, and oats 60). The exceptions were coconut (GI = 97) and rice (GI = 99) [36].

### 4.3. Unique Features of Some Plant-Based Beverages

For some, flax and hemp milks may be the beverages of choice because of their rich content of heart-healthy omega-3 fatty acids [37,38]. Quinoa-based beverages are new to the market. Quinoa has a good level of protein and a high quality of protein, [39]. Soy beverages are unique in their content of isoflavones. These soy phytochemicals have been shown to be protective against heart disease, breast and prostate cancer, and loss of bone mineral content [40]. The USDA database reports the isoflavone content of soymilk (fortified and unfortified) as 1.1–31.0 mg/100 g, with a mean value of 10.7 mg/100 g [41].

Rice and rice-based foods may contain a measurable amount of arsenic, a carcinogen [42]. Hence, it has been suggested to limit the consumption of rice products, such as rice milk, to no more than 1 to 3 servings a week [43]. Health authorities have recommended that children under the age of 5 should not have rice milk as part of their regular diet [44,45]. The analysis of 6 samples from 2 common brands of rice milk revealed arsenic levels ranging from 17 to 70 ppb (with an average of 30 ppb), levels that exceed the US drinking water upper limit of 10 ppb [44]. Brown rice syrup also contains arsenic, and has been used as a sweetening agent in some brands of hemp milk [44].

### 4.4. Sustainability Issues

An increasing number of consumers, especially among the younger generation, are concerned that their food choices be not only nutritious and healthy but also eco-friendly and sustainable [1]. Data on environmental sustainability of the beverages is not widely reported, and its influence on consumer beverage preference has not been reported. The sustainability issues of the plant-based dairy alternatives differ between the types of beverage and the issues vary in level of magnitude. Researchers at University of Oxford have reported that the production of plant-based beverages (such as oat, soy, almond and rice milk) are associated with only 22–38% of the greenhouse gas (GHG) emissions of the level associated with dairy milk production [46,47]. Water usage is also much less in the production of plant-based beverages than for dairy milk. While soy and oat production have a very low water usage, rice and almonds are quite water-intensive crops [47]. About 270 L of water are required for the production of one liter of rice milk, while 370 L are required for the production of one liter of almond milk [47]. Even so, almond and rice beverages still require much less water than the production of dairy milk. In addition, a glass of dairy milk requires nine times more land for its production than any of the dairy milk alternatives [47].

In addition to the heavy water usage in almond production [48], almond farming also has an adverse and sometimes lethal effect on bees used to pollinate the almond trees [49]. On the other hand, hazelnuts are pollinated by the wind rather than the honeybee, and grow in moist climates where water needs are much less of an issue. Rice is not only a high water consumer [47], it produces more GHG emissions than any other crop used for plant-based beverages [50]. The coconut tree impacts tropical biodiversity. It tops the list of oil-producing crops for the number of species threatened per million tons of oil produced [51]. Soy milk and oat milk are considered eco-friendly. Both are associated with low water and land usage and modest GHG emissions [47]. While almond, coconut, and rice based beverages have the environmental issues mentioned above, all plant-based dairy alternatives have markedly lower GHG emissions, water usage, and land usage associated with their production than dairy milk production. More research is needed for all the different types of non-dairy beverages to document their eco-friendliness and how they impact the health of the planet. We recognize that sustainability issues are very complex and there may be a great variability in the environmental impact not only between types of plant-based beverages but also within the same type, depending on the sustainability of farming techniques used for growing the crops. We have limited our discussion to some of the factors which are being used to market new products entering the market. For one such beverage, the company claims that it is incredibly good for the planet, using 74% less energy, 92% less water, and has 74% less CO_2_ emissions than regular milk [52].

Lastly, we recognize the limitations of our present research study and that we cannot generalize our findings to all 3 continents represented. This is just a snapshot of limited regions of the USA, Western Europe, and Australia. Each geographical area possibly contains hundreds of different beverages [8,12].

## 5. Conclusions

The rising popularity of a vegan lifestyle will continue to fuel the consumer demand for non-dairy plant-based beverages [1]. The plant-based beverages surveyed across 3 continents generally scored well in terms of containing modest levels of sodium and calories, and low levels of saturated fat. While beverages in US tended to have fewer calories, and European beverages tended to have lower sodium values, the differences were not dramatic. We found that pea-based beverages had the most sodium added, while coconut, hazelnut, and soy averaged the lowest levels of sodium. Furthermore, the grain beverages (such as rice and oats) had more sugar than other beverages types, while coconut, cashew, pea, and almond beverages had the lower sugar levels. All of the beverages had low levels of saturated fat with the exception of coconut beverages.

Almost 80% of the beverages were fortified with calcium, and 55% of the beverages were fortified to levels equal to or better than the calcium level of dairy milk. We found that almond, soy and pea-based beverages had the highest levels of calcium while coconut and cashew beverages had the lowest levels. The soy and pea protein-based beverages had 8–9 g protein/serving, comparable to the level in dairy milk, while rice and coconut-based beverages had less than 1 g of protein/serving.

Levels of vitamins D and B12 fortification were quite low. Since these beverages are significantly used by vegetarians, who are often marginal or deficient in vitamin B12, it is unfortunate that only 41% of all products examined had vitamin B12 fortification. Australian and European beverages had considerably lower levels of both vitamins D and B12. Among the beverages that were B12 fortified, we found rice beverages had the lowest content of vitamin B12, while soy, pea, cashew and almond beverages had the highest content of vitamin B12. For the beverages that were vitamin D fortified, almond and coconut beverages had the lowest content of vitamin D, while pea protein had the highest. Food manufacturers of plant-based beverages must be encouraged to better fortify their products with essential nutrients.

Many of the non-dairy plant-based beverages have significant health-promoting properties. Consumers need to be better informed regarding the nutritional content of non-dairy plant-based beverages as their nutrient profiles can vary greatly between the different types of beverages. Information about the health benefits of the beverages may also help consumers make healthier choices.

## Figures and Tables

**Table 1:** Percentages of non-dairy beverages that are fortified, and those meeting the 20% daily value level of fortification ^1^ (in brackets) per serving, by region.

	Combined 3 Regions	USA	Australia	Europe
*n*	148	60	48	40
Calcium	78 (70 ^2^)	87 (73)	79 (73)	63 (58 ^3^)
Vitamin D	53 (24 ^2^)	82 (47)	21 (8)	50 (8 ^3^)
Vitamin B12	41 (38 ^2^)	47 (40)	35 (35)	40 (38 ^3^)

^1^ For USA, the nutrition label uses the following daily values: calcium 1300 mg, vitamin D 20 mcg, vitamin B12 2.4 mcg. For Europe, the equivalent values used are calcium 1000 mg, vitamin D 15 mcg, vitamin B12 4 mcg. For Australia, the daily values used were 800 mg, 10 mcg, and 2.0 mcg, respectively. ^2^ using the 30% DRV value for Europe, the corresponding values for the combined regions would be 63, 22, and 34%, for calcium, D and B12, respectively. ^3^ using the European threshold of 30% DRV for a rich source (of vitamins and minerals), the % of beverages meeting that level of fortification would be 35, 0, and 23% for calcium, vitamin D, and vitamin B12, respectively. Serving size: 240 mL for USA; 250 mL for Australia and Europe.

**Table 2:** Median (Q1–Q3) values ^1^ of calcium, vitamin D and vitamin B12 levels used in fortification for fortified products, by world region.

	Combined 3 Regions		USA		Australia		Europe		*p* Value
	*n*	Median (Q1–Q3)	*n*	Median (Q1–Q3)	*n*	Median (Q1–Q3)	*n*	Median (Q1–Q3)	
Calcium	115	30 (22–37)	52	25 (20–35) ^a^	38	37 (27–38) ^a,b^	25	30 (22–30) ^b^	<0.001
Vitamin D	79	15 (10–25)	49	21.5 (10–25) ^a^	10	13 (12–37.5) ^b^	20	9 (9–12.5) ^a,b^	<0.001
Vitamin B12	61	50 (30–50)	28	50 (25–110) ^a^	17	50 (50–50) ^b^	16	36.5 (24–38) ^a,b^	<0.001

^1^ Values given as % daily value per serving (240 mL for USA; 250 mL for Australia and Europe). Kruskal–Wallis non-parametric test for independent samples with multiple pairwise comparisons were used to perform comparisons among regions. Different lowercase letters in the same row indicate significant differences among regions, after Bonferroni adjustment for multiple comparisons. *p* < 0.05 is considered statistically significant.

**Table 3:** Median (Q1–Q3) values of calories and selected nutrients in non-dairy beverages per serving, by region.

	Combined 3 Regions	USA	Australia	Europe	*p* Value
*n*	148	60	48	40	
Calories (kcal)	93 (63–125)	80 (50–115) ^a^	110 (76–142) ^a^	94 (67–123)	0.003
Sodium (mg)	100 (80–130)	105 (85–150)	108 (92–140) ^a^	100 (60–123) ^a^	0.037
Protein (g)	1.8 (1–5.8)	1.5 (1–5.5)	2.0 (1.4–7.9)	1.4 (0.8–3.9)	0.055
Saturated fat (g)	0.5 (0.2–1)	0 (0–1) ^a,b^	0.7 (0.5–1) ^a^	0.5 (0.3–0.9) ^b^	<0.001
Sugars (g)	4.6 (0.8–7.9)	4 (0–7)	4.3 (1.2–6.8)	6.6 (1.0–12.3)	0.056

Serving size: 240 mL for USA; 250 mL for Australia and Europe. Kruskal–Wallis non-parametric test for independent samples with multiple pairwise comparisons were used to perform comparisons among regions. Different lowercase letters in the same row indicate significant differences among regions, after Bonferroni adjustment for multiple comparisons. *p* < 0.05 is considered statistically significant.

**Table 4:** Percentage of non-dairy beverages meeting the suggested nutrient guideline per serving, by region.

	Combined 3 Regions	USA	Australia	Europe
*n*	148	60	48	40
At least 5 g protein	26	27	31	20
No more than 1 g saturated fat	87	88	85	88
No more than 5 g total sugars	55	60	60	43
No more than 120 calories	74	83	60	75
No more than 115 mg sodium	64	60	60	73
At least 1.5 g dietary fiber	23	18	25	28

Serving size: 240 mL for USA; 250 mL for Australia and Europe.

**Table 5:** Percentage of non-dairy beverages meeting the suggested nutrient guideline per serving, by type of beverage.

Type of Beverage	*n*	At Least 5 g Protein	No More than 1 g Saturated Fat	No More than 5 g Total Sugars	No More than 120 Calories	No More than 115 mg Sodium	At Least 1.5 g Dietary Fiber
Almond	33	0	100	64	97	55	9
Almond-coconut	6	0	67	67	100	67	0
Cashew	7	0	86	86	86	71	0
Coconut	10	0	0	80	100	80	10
Hazelnut	3	0	100	33	67	67	0
Macadamia	3	0	100	100	100	67	33
Oats	23	0	96	39	57	70	57
Pea	7	71	71	71	86	43	29
Rice	13	0	100	15	46	62	0
Soy	29	100	93	55	52	73	31
Legume + nuts/grains ^1^	6	83	100	67	67	17	17
Other beverages ^2^	8	13	88	50	75	86	25

^1^ Pea + oats; pea + almond + cashew; pea + almond (*n* = 2); soy + rice; pea + flax. ^2^ Rice + hazelnut; rice + coconut; oats + walnut; quinoa (*n* = 2); flax; hemp; walnut + almond + hazelnut. Serving size: 240 mL for USA; 250 mL for Australia and Europe.

**Table 6:** Median (Q1–Q3) values of calories and selected nutrients in non-dairy beverages per serving, by type of beverage.

Type of Beverage	*n*	Calories (kcal)	Sodium (mg)	Protein (g)	Saturated Fat (g)	Sugars (g)
Almond	33	66 (45–90) ^a,b,c^	117 (90–150)	1.3 (1–1.8) ^a,b,c^	0.3 (0–0.5) ^a,b,c^	3.6 (0.3–7)
Almond-coconut	6	52 (43–105)	104 (79–120)	1.2 (1–1.8) ^d^	1 (0.8–1.8) ^a^	0.9 (0–6)
Cashew	7	55 (48–77)	100 (95–115)	1.1 (0.7–1.7) ^e^	0.7 (0.3–1)	0.8 (0–3)
Coconut	10	57 (45–70) ^d,e,f^	85 (45–111)	0.1 (0–0.5) ^f,g,h,i^	4 (3–4.8) ^b,d,e,f,g^	1.8 (0–4.6)
Hazelnut	3	70 (50–125)	90 (70–112)	1 (1–1.8)	0.5 (0.3–0.6)	7.3 (3.6–7.5)
Macadamia	3	56 (53–65)	105 (103–139)	1 (0.9–1.1)	0.8 (0.8–0.9)	0.5 (0.3–2.4)
Oats	23	120 (112–141) ^a,d^	100 (91–120)	2 (2.0–2.5) ^f,j,k^	0.5 (0–0.8) ^d^	8 (4.5–12)
Pea	7	90 (81–100)	120 (98–179)	8 (4.5–8.9) ^a,g,l^	0.7 (0.6–1.9)	3 (0–4.5)
Rice	13	125 (120–137) ^b,e^	100 (86–163)	0.8 (0.3–1) ^j,l,m,n^	0.2 (0–0.3) ^e,h^	9 (7.1–11.8)
Soy	29	118 (99–142) ^c,f^	95 (76–123)	8.1 (7.8–9) ^b,d,e,h,k,m,o^	0.8 (0.5–1) ^c,h^	5.1 (1.8–7)
Legume + nuts/grains ^1^	6	98 (93–130)	190 (87–220)	9 (8–10) ^c,i,n^	0.5 (0–0.5) ^f^	2.5 (0.6–7)
Other beverages ^2^	8	65 (53–142)	75 (60–110)	1.4 (0.9–2.5) ^o^	0.1 (0–0.6) ^g^	5.5 (1.4–14)
	*p* value	<0.001	0.12	<0.001	<0.001	<0.001

^1^ Pea + oats; pea + almond + cashew; pea + almond (*n* = 2); soy + rice; pea + flax. ^2^ Rice + hazelnut; rice + coconut; oats + walnut; quinoa (*n* = 2); flax; hemp; walnut + almond + hazelnut. Serving size: 240 mL for USA; 250 mL for Australia and Europe. Kruskal–Wallis non-parametric test for independent samples with multiple pairwise comparisons were used to perform comparisons among regions. Different lowercase letters in the same column indicate significant differences among types of beverages, after Bonferroni adjustment for multiple comparisons. *p* < 0.05 is considered statistically significant.

**Table 7:** Median (Q1–Q3) of calcium, vitamin D and vitamin B12 levels (%daily value) for only fortified beverages per serving, by type of beverage.

Fortified Beverages						
	Calcium		Vitamin D		Vitamin B12	
Type of Beverage	*n*	Median (Q1–Q3)	*n*	Median (Q1–Q3)	*n*	Median (Q1–Q3)
Almond	24	35 (25–38)	12	12 (10–25)	7	50 (31–50)
Almond-coconut	4	34 (27.5–36)	2	25 (17–37.5)	2	44 (38–50)
Cashew	5	16 (4–37)	3	17.5 (9.5–25)	3	50 (44–85)
Coconut	8	16 (8–32.5)	7	11.2 (10–25)	6	37 (30–50)
Hazelnut	1	23 (–)	1	9 (–)	1	38 (–)
Macadamia	2	21.5 (8–35)	1	20 (–)	1	240 (–)
Oats	19	25 (22–35)	14	15 (12.5–20)	10	39 (24–50)
Pea	7	30 (24.5–39)	6	25 (23–25)	5	35 (25–35)
Rice	10	25 (23.5–32)	6	15 (12–20)	6	25 (25–25)
Soy	25	30 (22–37)	16	15 (12.5–20)	18	50 (50–60)
Legume + nuts/grains ^1^	5	30 (25–37)	5	17.5 (10–25)	1	55 (50–60)
Other beverages ^2^	5	20 (20–30)	5	10 (10–25)	1	60 (−)
*p* value	0.231		0.735		0.022	

^1^ Pea + oats; pea + almond + cashew; pea + almond (*n* = 2); soy + rice; pea + flax. ^2^ Rice + hazelnut; rice + coconut; oats + walnut; quinoa (*n* = 2); flax; hemp; walnut + almond + hazelnut. Serving size: 240 mL for USA; 250 mL for Australia and Europe. Kruskal–Wallis non-parametric test for independent samples with multiple pairwise comparisons were used to perform comparisons among regions. After Bonferroni adjustment, no statistical differences among groups was detected. *p* < 0.05 is considered statistically significant.

## Data Availability

The data presented in this study are available on request from the corresponding author.

## Supplementary Materials

The following are available online at https://www.mdpi.com/2072-6643/13/3/842/s1, Table S1: Median (Q1–Q3) values of selected micronutrients used in fortification for all non-dairy beverages (both fortified and non-fortified), by world region title; Table S2: Median (Q1–Q3) of calcium, vitamin D and vitamin B12 levels (%daily value) for all non-dairy beverages (both fortified and non-fortified) per serving, by type of beverage.
