# Supplementary material for: International Analysis of the Nutritional Content and a Review of Health Benefits of Non-Dairy Plant-Based Beverages
Source: Nutrients. 2021 Mar 4;13(3):842. doi: 10.3390/nu13030842 (PMC7999853; doi:10.3390/nu13030842)
Supplement: Supplementary file 1 [file nutrients-13-00842-s001.pdf]

Table S1. Median (Q1-Q3) values[1] of selected micronutrients used in fortification for all non-dairy beverages (both fortified and non-fortified), by world region.

	Combined 3 regions	USA	Australia	Europe	
n	148	60	48	40	p value
Calcium	25 (7-35)	25 (10-35)	37 (13-38)[a]	22 (0-30)[a]	<0.001
Vitamin D	8.5 (0-15)	15 (10-25)[a,b]	0 (0-0)[a]	3 (0-9)[b]	<0.001
Vitamin B12	0 (0-38)	0 (0-50)	0 (0-50)	0 (0-24)	0.234

[1] Values given as % daily value per serving (240ml for USA; 250ml for Australia and Europe).
Kruskal–Wallis non-parametric test for independent samples with multiple pairwise comparisons were used to perform comparisons among regions. Different lowercase letters in the same row indicate significant differences among regions, after Bonferroni adjustment for multiple comparisons. P<0.05 is considered statistically significant.

Table S2. Median (Q1-Q3) of calcium, vitamin D and vitamin B12 levels (%daily value) for all non-dairy beverages (both fortified and non-fortified) per serving, by type of beverage

		All beverages		
**Type of beverage**	n	Calcium	Vitamin D	Vitamin B12
Almond	33	30 (2-37)	0 (0-10)	0 (0-0)
Almond-coconut	6	27.5 (0-35)	4.5 (0-25)	0 (0-38)
Cashew	7	10 (3-29.5)	9 (0-17.5)	0 (0-44)
Coconut	10	10 (4-30)	9.5 (0-12.5)	27 (0-38)
Hazelnut	3	0 (0-11.5)	0 (0-4.5)	0 (0-19)
Macadamia	3	8 (4-21.5)	0 (0-10)	0 (0-120)
Oats	23	25 (20-35)	10 (0-17.5)	0 (0-31.5)
Pea	7	30 (24-39)	23 (11.5-25)	25 (12-35)
Rice	13	25 (20-30)	0 (0-12.5)	0 (0-25)
Soy	29	30 (19-37)	9.5 (0-15)	44 0-50)
Legume + nuts/grains[1]	6	27.5 (25-37)	10 (0-25)	0 (0-50)
Other beverages[2]	8	17.5 (0-25)	8 (0-17.5)	0 (0-0)
	p value	0.185	0.735	0.075

[1] Pea+ oats; pea+almond+cashew; pea+almond (n=2); soy+rice; pea + flax
[2] Rice+hazelnut; rice+coconut; oats+walnut; quinoa (n=2); flax; hemp; walnut + almond + hazelnut
Serving size: 240ml for USA; 250ml for Australia and Europe. Kruskal–Wallis non-parametric test for independent samples with multiple pairwise comparisons were used to perform comparisons among regions. P<0.05 is considered statistically significant.
